# ContigScape: a Cytoscape plugin facilitating microbial genome gap closing

**DOI:** 10.1186/1471-2164-14-289

**Published:** 2013-04-30

**Authors:** Biao Tang, Qi Wang, Minjun Yang, Feng Xie, Yongqiang Zhu, Ying Zhuo, Shengyue Wang, Hong Gao, Xiaoming Ding, Lixin Zhang, Guoping Zhao, Huajun Zheng

**Affiliations:** 1 State Key Laboratory of Genetic Engineering, Department of Microbiology, School of Life Sciences, Fudan University, Shanghai, 200433, China; 2 Shanghai-MOST Key Laboratory of Health and Disease Genomics, Chinese National Human Genome Center at Shanghai, Shanghai, 201203, China; 3 CAS Key Laboratory of Pathogenic Microbiology & Immunology, Institute of Microbiology, Chinese Academy of Sciences, Beijing, 100190, China; 4 CAS Key Laboratory of Synthetic Biology, Institute of Plant Physiology and Ecology, Shanghai Institutes for Biological Sciences, Chinese Academy of Sciences, Shanghai, 200032, China; 5 Department of Microbiology and Li KaShing Institute of Health SciencesThe Chinese University of Hong Kong, Prince of Wales Hospital, Shatin, New Territories, Hong Kong SAR, China; 6 Graduate School of Chinese Academy of Sciences, Beijing, 100049, China

**Keywords:** ContigScape, Repeat contig, Microbial, Visualization, Linkage, Gap closing

## Abstract

**Background:**

With the emergence of next-generation sequencing, the availability of prokaryotic genome sequences is expanding rapidly. A total of 5,276 genomes have been released since 2008, yet only 1,692 genomes were complete. The final phase of microbial genome sequencing, particularly gap closing, is frequently the rate-limiting step either because of complex genomic structures that cause sequence bias even with high genomic coverage, or the presence of repeat sequences that may cause gaps in assembly.

**Results:**

We have developed a Cytoscape plugin to facilitate gap closing for high-throughput sequencing data from microbial genomes. This plugin is capable of interactively displaying the relationships among genomic contigs derived from various sequencing formats. The sequence contigs of plasmids and special repeats (IS elements, ribosomal RNAs, terminal repeats, *etc.*) can be displayed as well.

**Conclusions:**

Displaying relationships between contigs using graphs in Cytoscape rather than tables provides a more straightforward visual representation. This will facilitate a faster and more precise determination of the linkages among contigs and greatly improve the efficiency of gap closing.

## Background

The emergence of next-generation sequencing (NGS) technology greatly facilitated genome sequencing. The long reads produced by Roche 454 or PacBio SMRT makes *de novo* assembly easier to complete. Despite the symmetrical representation of sequences produced by 454 or other NGS methods, tens to hundreds of contigs still exist due to repeat sequences or GC/AT-rich regions in the genomes. Therefore, determining the order of contigs and filling in the gaps among them using PCR are two essential and rate-limiting steps in the final phase of whole-genome sequencing. The ‘Newbler Assembler’ developed by Roche 454 has strict parameters to avoid mis-assembly and thus results in the breakdown of some contigs. For example, one read would be separated and placed into two contigs due to base-calling variation in different reads, and in some extreme cases, no gap truly existed between two such “contigs”. Several existing scaffolders for high throughput sequencing (HTS) genome assemblies, such as GRASS [1], SSPACE [2], OPERA [3] and MIP Scaffolder [4], may provide effective scaffolding; however, they lack global visualization and have to face the balance between scaffold length and accuracy. Most visualization tools, such as Consed [5], DNASTAR lasergene [6] and Gap [7], which are often used for genome completion and enable users to verify the assembly of contigs, can only display a linear relationship of contigs [8]. To provide a genome-level overview, ABySS-Explorer [9] and TGNet [10] were developed. TGNet incorporates several scripts for converting transcripts to facilitate assembly and represents contigs graphically using points. ABySS-Explorer [9] is another global viewer of contig assembly. However, neither program was designed to treat repeat contigs or display the reads that link contigs and imply the location of gaps and repeat contigs [8,10] (Table 1). These programs also lack special functions for microbial genome analysis. Therefore, we developed ContigScape, a Cytoscape [11] plugin that can be used to display all relationships of contigs, including each contig and linked reads in a microbial genome; the gaps and repetitive sequences can then be confirmed by users. Our goal is to display the original relationships of all contigs instead of a manually trimmed result, as the real association of contigs should be depicted as a network rather than a linear linkage. Furthermore, repeat contigs, gaps and even plasmids can be highlighted, filtered, and customized.

**Table 1:** Comparison to other genomic display tools

**Program**	**Main display**	**Connections between contigs**	**Connections between scaffolds**	**Compatible assemblers**	**Objects**	**Global contig display**	**The weight of contigs’ relationship**	**Type**
**Consed**	Linear	From paired reads	No	Any producing ACE files	nonselective	No	No	stand-alone
(Gordon et al., [5])								
**Phrapview**	Linear	From paired reads	No	Phrap	nonselective	No	No	stand-alone
(Gordon et al., [5])								
**Gap5**	Linear	No	No	All	nonselective	No	No	stand-alone
(Bonfield et al., [12])								
UCSC	Linear	No	No	No	nonselective	No	No	stand-alone
(Kent et al., [13])								
**Ensembl**	Linear	No	No	No	nonselective	No	No	stand-alone
(Stalker et al., [14])								
**IGV**	Linear	No	No	All	nonselective	No	No	stand-alone
(Robinson et al., [15])								
**EagleView**	Linear	No	No	Any producing ACE files	nonselective	No	No	stand-alone
(Huang, Marth [16])								
**Hawkeye**	Linear	From paired reads viewed within a single scaffold	No	Any producing AFG files	nonselective	No	No	stand-alone
(Schatz et al., [17])								
**ABySS-Explorer**	Graphs	From paired reads	No	ABySS	nonselective	One node	No	stand-alone
(Nielsen et al., [9])								
**TGNet**	Graphs	From transcripts, From scaffolding information	From transcripts	All	eukaryocyte	One node	No	Perl scripts
(Oksana et al., [10])								
**ContigScape**	Graphs	From reference, From scaffolding information, 454 repeat reads or other database	From reference, From other database	All	Fungi,,bacteria, plasmid, virus *etc.*	One edge and two nodes	display	plugin
**(**the publication**)**								

ContigScape is a convenient Java plugin based on Cytoscape [11], which is an established, free, and open-source software platform for the visualization and analysis of molecular interaction networks and can be used on Windows, Linux and Mac platforms. ContigScape is a simple and efficient plugin that makes gap closing during microbial genome sequencing more efficient.

## Implementation

### Sequencing of samples, de novo assembly of the genomes, and scaffolding

All genome sequences used in Table 2 had been released in GenBank and were generated by different laboratories in China and sequenced by the Chinese National Human Genome Center ast Shanghai. In our approach, genome sequencing was conducted using the Roche 454 GS FLX system and the GS FLX Titanium Sequencing Kit. Reads were then *de novo* assembled using Newbler v2.3. We constructed the mate-pair DNA libraries with insert sizes larger than 3 kb and sequenced using the Illumina Hiseq 2000 sequencing platform. A random subset of mate-pair reads were used for mapping and analysis with scaffold.pl (perl script, see Additional file 1, using BWA [18], Samtools [19], FASTX-Toolkit and BEDTools [20] programs).

**Table 2:** Strains used in this study and general sequence information

**ID**	**Type**	**S`trains**	**Size (Mbp)**	**Repeat contigs**	**All contigs**	**Large contigs**	**Coverage**	**Average length**	**Ribosomal RNAs**	**Plasmid**	**Accession number**
**1**	bacteria	*Amycolatopsis mediterranei* S699 [21]	10.25	16	75	67	31	532	4	none	CP003729
**2**	bacteria	*Ralstonia solanacearum* Po82 [22]	3.48, 1.95	26	149	115	27	328	3	none	CP002819-CP002820
**3**	bacteria	*Amycolatopsis orientalis* HCCB10007	8.95	14	69	53	25	408	4	1	CP003410
**4**	bacteria	*Mycobacterium tuberculosis* CCDC5079	4.41	27	179	147	29	434	1	none	CP002884
**5**	bacteria	*Leptospirillum ferriphilum* ML-04 [23]	2.41	>50	267	213	31	311	2	none	CP002919
**6**	bacteria	*Bacillus thuringiensis* BMB171 [24]	5.64	10	221	168	32	391	14	1	CP001903-CP001904
**7**	bacteria	*Edwardsiella tarda* EIB202 [25]	3.76	15	223	64	17	256	7	1	CP001135
**8**	archaea	*Acidianus hospitalis* W1 [26]	2.16	1	11	7	31	409	1	1 integrated	CP002535
**9**	virus	*Cotesia vestalis*	0.52	>35	572	265	135	381	none	none	HQ009524-HQ009558
		*Bracovirus*[27]									
**10**	mycoplasma	*Mycoplasma bovis*	0.95	25	111	75	49	360	2	none	CP002513
		Hubei-1 [28]									
**11**	fungi	*Cordyceps militaris*[29]	32.2	>100	2426	1670	147	385	NA	none	AEVU00000000

### Programming language, systems, and external programs

ContigScape was developed based on Cytoscape, which is available for Linux, Windows and MacOS X. The core programming language of ContigScape is Java. Users are provided with a comprehensive manual that explains all functions (see Additional file 1).

### Counting contig abundance and copy number, and display

**Figure 1:**
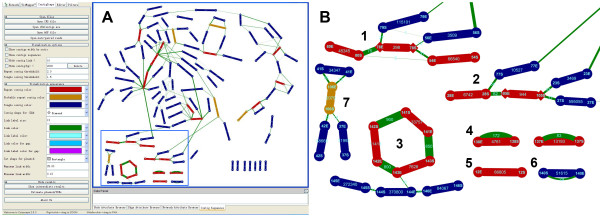
**A sample genome displayed by ContigScape. A**, the ContigScape interface. The left portion of the screen represents the control panel. The window on the right shows a sample genome. Contigs are colored red (repeat contig), dark blue (unique contig) and orange (probable repeats). **B**, a zoomed image of some contigs. Some contigs (light blue frame in **A**) were zoomed to present an enlarged image. B1. A linear plasmid formed by three contigs. B2. Repeats (Contig28) at the end of the chromosome. B3. A circular plasmid with high copy number formed by three contigs (Contig141, 142, and 143). B4. Two high-copy-number circular plasmids each formed by a single contig. B5. A linear plasmid with high copy number was formed by one contig. B6. A circular plasmid with single copy number composed of one contig.

Our interest lies in estimating the abundance of repeat contigs. We define a repeat contig as one at least having twice as much read coverage than the average genome coverage. Average genome coverage is the ratio of the total bases of reads assembled into contigs and the total size of all contigs. When users input Contig Relationship Scape (CRS) file in our plugin without original assembly result, the default arithmetic for genome coverage is to count the average coverage of all contigs with size bigger than 20 kb (In our experience, the repeat contig bigger than 20 kb is rare in microbial genome except plasmid). Each copy number is calculated as the ratio of contig abundance and average genomic coverage, which represents the corresponding repetition rate of the contigs. Contig abundance is the ratio of total bases of reads assembled into this contig and the contig size. We define a specific contig as one having read coverage less than 1.5 fold average (default value is 1.5, which can be set by users). So, the contigs whose coverage is greater than 1.5 and less than 2 are probable repeats. They need to be confirmed by counting the connections at the end of the contig or PCR method. Like Figure 1B7, 106S-106E is a repeat contig verified by two linkages in each end. PCR needs to be used to determine the relationship “37S-37E-106S-106E-41S-41E-106E-106S-42E-42S” or “37S-37E-106S-106E-41E-41S-106E-106S-42E-42S”.

Meanwhile, the average number of linkages between contigs can be computed by $Z\;=\;\sum_{1}^{n}\mathrm{linkNum}\;\mathrm{between}\;\mathrm{largecontigs}\;\left(\mathrm{size}\;\geq \;20\mathrm{Kb}\right)/n$, where Z is the average number of linkages and n is the number of relationships conforming to the requirements. As above, the ratio of link number and Z indicates the width of edge representing linkage in CytoScape.

### Principles of displaying Roche 454 genome assembly results

**Figure 2:**
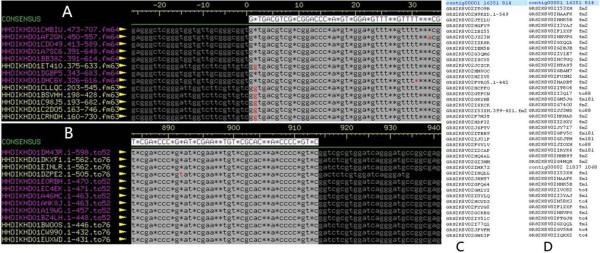
**A 913-bp repeat contig assembled with 454 reads. A**. The 5 prime end of the contig, independently assembled by the reads from Contig64 and Contig63. **B**. The3 prime end of the contig, assembled as described in panel A, but reads extended into Contig52 and Contig76. **C**. The list of read names from the “ace” file. **D**. The list of reads whose names contained ‘fm’ or ‘to’, which linked to the unique and repeat contigs, respectively.

**Figure 3:**
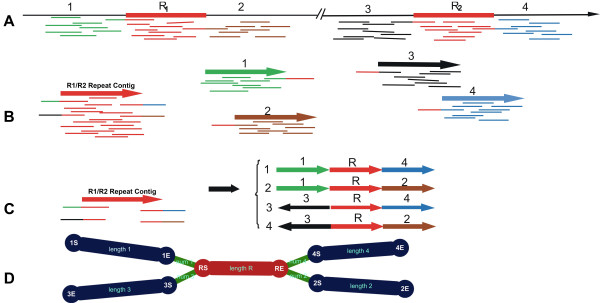
**Schematic diagram of assembly from 454 reads and the relationship of repeat contigs. A**. The genome has four unique sections (1–4) and two repeats (R1 and R2). **B**. One repeat contig and four unique contigs were assembled. The reads coming from R1 and R2 was assembled into the same contig, resulting in twice the coverage of other contigs. Some reads at the end of the repeat contig consisted of only partial sequences, and the other parts of the reads are located in other contigs. **C**. We can obtain four linkage relationships of the repeat contigs depending on reads covering different contigs. Among them 2 and 3 reflect the correct linkage whereas 1 and 4 was incorrect. **D**. The relationship shown in C was displayed in ContigScape. 1S-1E represent contig1; 2S-2E represent contig2; 3S-3E represent contig3; 4S-4E represent contig4; RS-RE represent contigR; red coloring represents repeat contigs, dark blue coloring represents unique contigs. “S” represents the starting position of the contig and “E” represents the termination location of the contig. Num 1, Num 2, Num 3, Num 4 represent the number of reads connecting contig R and “1E”, “2S”, “3S”, and “4S”, respectively. Length1, length2, length3 and length4 represent the lengths of contig1, contig2, contig3 and contig4, respectively. The width of the green edge is proportional to their number.

Roche 454 reads now exceed 700 base pairs in length and thus can be used to resolve gaps caused by small repeats. The ‘Newbler Assembler’ may produce a ‘454Contigs.ace’ file, which contains all assembly information and can be shown by ‘Consed’ [5]. As indicated in Supporting Figure 2, when a read was separated into two contigs, the coordinate of the read in each contig was shown after the read name, followed by the contig number with which this read was linked. The general principle to label the reads spanning the linked contigs is to use ‘fmX’ to represent the 5’ end of the reads located in contigX and ‘toY’ to represent the 3’ end of the reads located in contigY. This unique feature of the ‘Newbler Assembler’ labeling system in conjunction with long reads from 454 enables us to extract all the information of ‘fm’ and ‘to’ from the ‘454Contigs.ace’ file. This information can then be arranged into a relationship table (Figure 2C, D), such as ‘5’-end-Contig1’ linked to ‘3’-end-Contig2’. This relationship table can then be displayed by ContigScape as shown in Figure 3D.

### Principles of displaying scaffolds constructed by mate-pair reads

**Figure 4:**
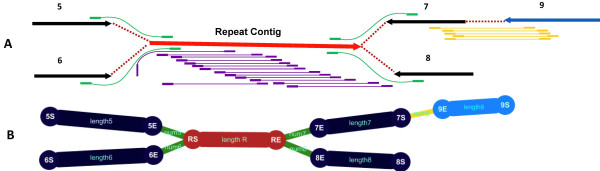
**Schematic map of scaffolding. A**. Red arrows represent repeat contigs, and black and blue arrows represent unique contigs, where the orientation of the arrow represents the direction from the 5’ to 3’ end. The purple lines represent mate-pair reads within contigs whereas green and yellow lines represent mate-pair reads spanning contigs. The mate-pair reads represented by yellow lines are mapped into unique contigs and thus they can form a scaffold. Mate-pair reads represented by green lines failed to construct scaffolds because one end of these mate-pair reads is located in repeat contigs. **B**. Using ContigScape to describe graph A.

A scaffold is a consensus sequence formed by ordered contigs using ‘N’ to fill any gaps. The most common method uses the mate-pair information to assemble contigs into scaffolds. Scaffolding programs can determine the separation of two contigs depending on the fragment size of the mate-pair reads. For example, if two contigs were separately mapped by a pair of 3-kb mate-pair reads, the two contigs could be joined into a scaffold, and the gap size would be 3 kb minus the distance between the mapping loci and the end of contigs. This method would allow repeat regions less than 3 kb in length to be bridged. However, ambiguous linkages can occur if the repeat region was longer than the fragment size of the mate-pair library (Figure 4). Similar to the results from ‘Newbler’ for 454 reads, ContigScape can display a relationship network within scaffolds by counting the number of mate-pair reads linking to large contigs (>500 bp).

## Results and discussion

### Visualization

Repeats are usually assembled into single contigs and thus cause gaps. After sequencing, two repeat regions (R1 and R2, Figure 3A) were assembled into the R1/R2 repeat contig (Figure 3B), and ContigScape reported all of its possible linkages with other regions (1–4, Figure 3C–D). Further PCR validation guided by this predicted linkage would exclude the incorrect relationships and result in a final correct consensus sequence. The repeat contigs in ContigScape are shown in red (Figure 3D) to distinguish them from the normal contigs shown in dark blue (default setting). In addition, the number of reads connecting two contigs is labeled with linkage edges, and the linkage reliability is illustrated by variable edge thickness.

The key feature of ContigScape is to determine the linkage of two contigs assembled from 454 or Illumina reads. An ‘Ace’ file can be opened directly by ContigScape and the relationship of contigs can be saved as a CRS format (see sample, tabbed.txt, tabbedCov.txt, Additional file 1). The CRS format includes two files, and each contains three columns. ‘tabbed.txt’ contains the number of connections among contigs, and ‘tabbedCov.txt’ describes the length and coverage of contigs. The ‘tabbed.txt’ is similar to AGP file and describes how the chromosomes and scaffolds were assembled from the component contigs, but does not require contigs to be sorted in advance. It will produce an original graph after loading the two files, and a final graph needed for the layout function of Cytoscape. Researchers can also obtain the CRS information by converting the results from GRASS, SSPACE, OPERA and MIP scaffolders.

Another prominent characteristic of ContigScape is the calculation of the coverage of contigs and the subsequent definition of the contig whose coverage exceeded two fold above the average, denoted as ‘repeat contig’. Each contig is represented by one edge and two nodes, with ‘XS’ and ‘XE’ indicating the 5’ end (Start) and 3’ end (End) of contigX (X represents a number), respectively. The linkage (reads) is represented by a sole edge whose thickness varies based on the number of supporting reads. The number on the edge of contigs indicates the contig length, whereas the number on the edge of linkages indicates the number of linking reads.

### Application of technology to display 454 contigs and scaffolding by mate-pair reads

**Figure 5:**
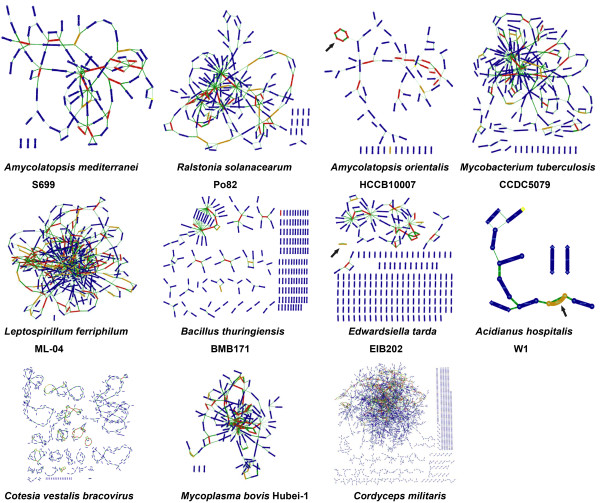
Contigs’ network of eleven 454Contigs.ace files.

**Figure 6:**
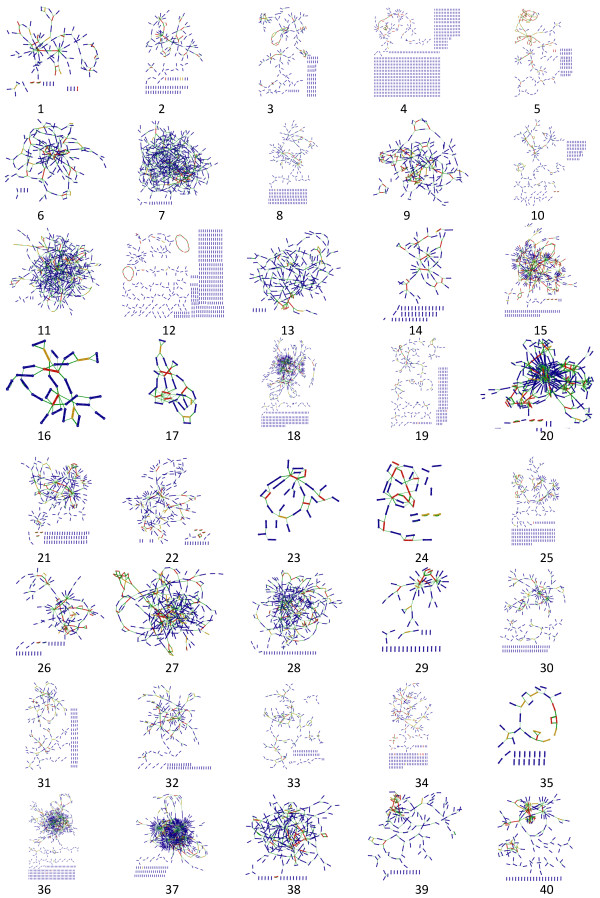
**The contig network of 40 strains using the 454Allcontigs.ace file.** This figure includes 19 genus strains: 1–11 are *Streptomyces,* 12 is *Penicillium,*13 is *Actinoplanes,* 14 is *Amycolatopsis,* 15 is *Bacillus,* 16*,*17 is *Brucella*, 18,36,37 are *Ralstonia,* 19 is *Burkholderia,* 20*–*22 are *Escherichia,* 23*,*24 are *Ketogulonicigenium,* 25 is *Klebsiella,* 26 is *Lactobacillus,* 27*,*28 are *Leptospira,* 29 is *Lysinibacillus,* 30*–*34 are *Mycobacterium,* 35 is *Mycoplasma,* 38 is *Rhizobiales,* 39*,*40 are *Vibrio.*

We have used this tool for the visualization of eleven genomes (Table 2, Figure 5), accelerating the completion of these genomes (nine of them have been published). After *de novo* assembly by 454 Newbler, researchers can estimate the complexity of specific genomes and the difficulty of gap closing with global views. In Figure 5, we see significant differences in the assembly of eleven genomes due to variance in the number of total contigs and repeat contigs. In addition, ContigScape has been applied to gap closing of an additional 40 genomes (Figure 6); the network of contigs in *Streptomyces*, *Leptospira* and *Ralstonia* is complex, whereas the contig graphs of *Brucella*, *Mycoplasma* and *Ketogulonicigeniumis* is simple. These genomes comprised bacteria, archaea, virus and fungi. It was clear that the gap closing for *A. hospitalis* W1 was easy. In the graph of *A. hospitalis* W1, we saw that the 28-kb contig3 was a tandem repeat, which had previously been identified as an integrated plasmid [26]. It is easy to determine if the plasmid is circular and if the copy number exceeds two, such as *A. orientalis* HCCB10007 and *E.tarda* EIB202 [25]. The 24th graph of Figure 6 shows four circular plasmids composed of only one contig. There was also a high-copy-number contig in the graph of *Mycobacterium tuberculosis* CCDC5079, and BLAST identified it as IS*6110*, an insertion element. The 14 rRNA operons of *Bacillus thuringiensis* BMB171 [24], each of approximately 5 kb in length, can also be clearly displayed (Figure 5). There were also many independent and closed rings in the assembly graph of *Cotesia vestalis Bracovirus*[27], which were identified as 35 non-redundant circular genome segments. The number of contigs in the fungus *Cordyceps militaris*[29] exceeded 2,000, therefore the contigs need further scaffolding.

We applied ContigScape to a recently assembled *Streptomyces sp* genome with 111 contigs sequenced by Roche 454 without scaffolding. We added seven contigs (contig140, 141, 142, 143, 144, 145 and 146) into the two CRS files to show different plasmids (Figure 1B). After processing, we found 25 repeat contigs, constituting six plasmids, 8 rRNA operons and one telomere (contig28, Figure 1B2). The remaining repeats include IS elements, phage or other sequences. Figure 1A shows that 52 nodes have no linkage, and they need additional scaffolding information. Therefore, PCR is necessary to fill the remaining gaps. Any relationships requiring validation are indicated by a green edge.

Judging whether a repeat contig was from chromosome or plasmid mainly depended on the linkage information of two ends of this contig. Four different types were shown in Figure 1B: 1). Repeat contigs connected in a circular fashion (Panel 3), 2). Individual contig connected itself without anyone else (Panel 4 and 6), 3). One end of repeat contig having no linkage to any other contigs, usually representing linear chromosome telomere or linear plasmid end (Panel 1 and 2), 4). A linear plasmid composed of only one repeat contig without connections to any contigs (Panel 5). While if a plasmid is linear and single copy, ContigScape cannot distinguish it. We can estimate whether or not a contig was a plasmid effectively based on above described situation in our experience. Of course researcher must confirm whether it is a plasmid or not by PCR, sequencing and annotation.

In Figure 1B, 143E has connections with 142E and 144E (Panel 3). But the number of connections (800) between 143E and 142E is more than that (10) between 143E and 144E. In this case, the latter might be a nonspecific connection caused by little overlap among the reads. Additionally, Figure 1B shows that contig78 in the linear plasmid 80E-80S-78E-78S-54E-54S also has another copy in the chromosome (Panel 1).

**Figure 7:**
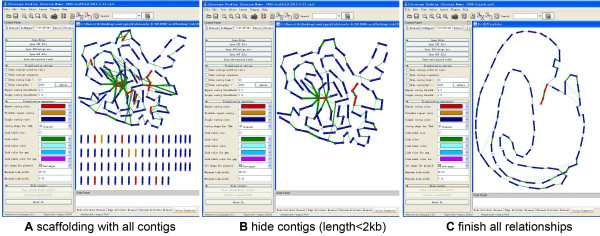
**The ContigScape interface and displaying connections between contigs. A**. The scaffolding of the 454LargeContigs of the *Streptomyces* genome using mate-pair libraries is shown. **B**. Hiding the contigs smaller than 2 kb in length and determining the linkages between the remaining specific contigs. **C**. Completing all linkages by reference, PCR, and other databases, and then obtaining the linear chromosomal sequence with terminal inverted repeats formed by two repeat contigs.

We also applied this program to another *Streptomyces sp* genome with 145 contigs sequenced by Roche 454 with mate-pair information (Figure 7). We can better interpret the relationship between contigs by using mate-pair reads. Figure 7C represents a linear chromosome with an 18 kb repeat at the ends (telomeres).

### Display functionality of ContigScape

**Figure 8:**
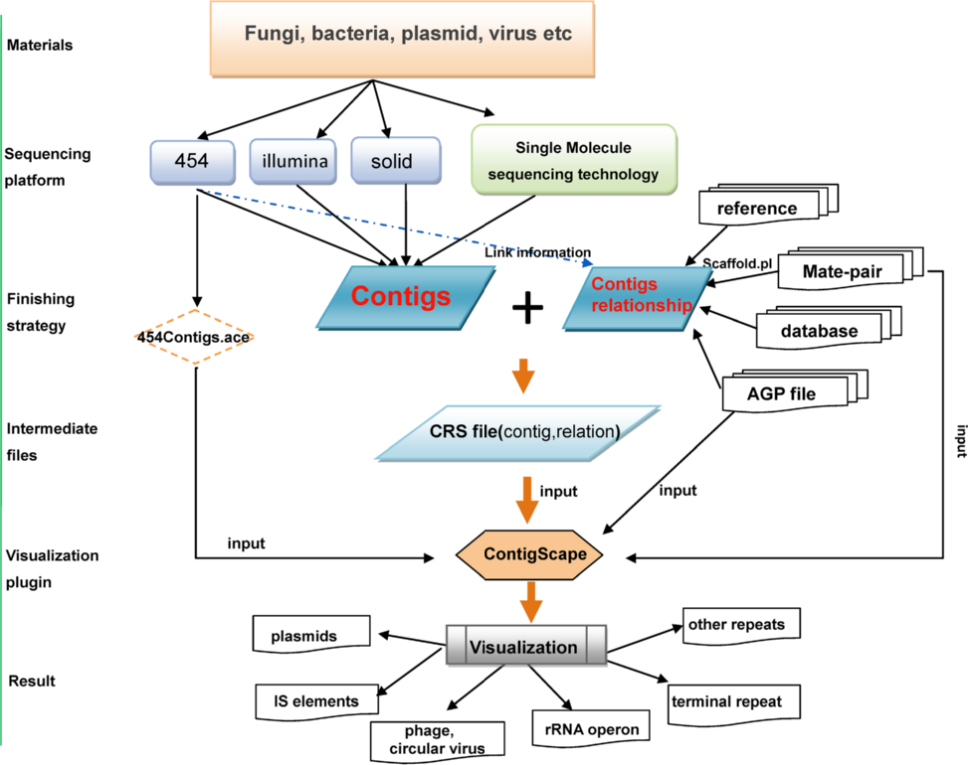
Workflow of visual strategy.

There are several unique features of ContigScape for microbial genome analysis (Figure 1). In particular, the “find genomic features” function may identify contigs belonging to plasmid/terminal repeats, determine whether the plasmid was linear or circular, and counting the read coverage of this plasmid (Figure 1B). Second, ContigScape may determine the locations of the ends of linear chromosomes based on a repeat contig where in one end has two edges and the other has none. After the ‘Ace’ file is loaded, the genomic structure network can be displayed, including the linkage of contigs, contig size and number of repeats. Meanwhile, another plugin called Network Analyzer [30] can be used to determine the complexity of the network (genome), and thus estimate the amount of work required to complete the genome. When viewing the graph, the 1,000 base pairs of both 5’-end and 3’-end can be loaded, with 20 “N” linking them representing the middle sequences. Clicking the edge of two contigs, the sequence containing corresponding contigs’ ends can also be displayed. The displayed sequence can be used to design primers in ContigScape and perform blast against NCBI database. In addition, the user can open “edit panel” to edit the connections of the network. In addition to gap closing in bacterial genomes, complete BAC or plasmid sequences can also be finished using ContigScape. It can also display if a CRS file, converted from scaffolding results using different methods, was imported. The workflow of ContigScape is shown in Figure 8. Other functions of ContigScape are described in an Additional file 1 (see ContigScape manual).

## Discussion

Comparative assembly [31] utilizes a reference genome sequence as a guide to discern repeat contigs. However, there are three obvious weaknesses regarding comparative assembly: (1) the target species must have previously been sequenced and assembled; (2) structural variations exists in different references; (3) it cannot resolve large insertions. For example, we resequenced *Amycolatopsis mediterranei* S699 and assembled the genome *de novo*[21]. Comparing with the previously released *A. mediterranei* S699 assembly [32], which was assembled using *A. mediterranei* U32 as a reference, the genome we sequenced contained a 10-kb insertion. The differences can likely be attributed to the different strategies used for genome assembly [21]. *De novo* assembly is a reliable way to avoid these weaknesses of comparative assembly.

Each sequencing technology has its own biases that result in coverage gaps. As coverage increases, the number of gaps decreases. However, gaps can occur if reads that would typically be assembled into one contig cannot span a large repeat area. Therefore, utilizing repeat contigs is important. During scaffold construction, repeat contigs usually cause errors in scaffolding or in the creation of linkages. Some programs may elect to link two unique contigs with one repeat contig, thus the individual repeat contig is used only once. Therefore, correct judgment will greatly reduce the efforts invested in genome assembly. Displaying straightforward graph-based relationships of contigs in Cytoscape rather than tables also facilitates a faster and more precise determination of the linkages among contigs. Our goal is to display the original relationships of all contigs rather than the manually trimmed results because the true association of contigs should be depicted as a network rather than a linear linkage.

ContigScape isn’t an assembly program and cannot replace phred/phrap/consed package, indeed they are complementary to each other. Consed [33] and its process “autofinish” [34] are very useful in gap closing. Actually, all contigs’ PHD files together with ABI3730 data sequenced after PCR must be assembled using phrap and edited by consed at last in our finishing strategy. ContigScape looks like a canvas used to judge and edit the order among contigs and can evaluate the complexity of shot-gun assembly in global visually. The plugin can only process several NGS assembly data directly like 454Conitgs.ace and mate-pair reads, while the assembly result made by other programs should be transformed into CRS file as input.

## Conclusions

Using ContigScape, contigs can be displayed and repeat contigs, gaps, and even plasmids can be highlighted, filtered, and customized. We designed unique functions for microbial genome analysis in ContigScape, such as the identification of plasmids, whether they are linear or circular and an estimation of their read coverage. We believe with the development of the third-generation sequencing technologies, gap closing will be much easier due to fewer assembled contigs. Long repeats will still hamper the assembly, especially in larger genomes; however, ContigScape will play an important role in gap closing for these genomes.

## Accession numbers

The genome sequences have been deposited at NCBI under the accession numbers:

[GenBank: CP003729], [GenBank: CP002819], [GenBank: CP002820], [GenBank: CP003410], [GenBank: CP002884], [GenBank: CP002919], [GenBank: CP001903], [GenBank: CP001904], [GenBank: CP001135], [GenBank: CP002535], [GenBank: HQ009524-HQ009558], [GenBank: CP002513], [GenBank: AEVU00000000].

## Availability and requirements

**Project name**: ContigScape

**Project home page**: http://sourceforge.net/projects/contigscape/.

**Operating systems**: Windows, Linux, MacOSX.

**Programming language**: Java, Perl

**Software packages (Linux)**: Fastx_toolkit 0.0.13, BEDTools 2.14.3, BWA 0.5.7, Samtools 0.1.18

**Other requirements**: Java 1.6 or higher, Cytoscape 2.8.3 (After Java and Cytoscape are installed, put ContigScape.jar under cytoscape2.8.3/plugins folder).

**License**: GNU

**Restriction for non-academics**: Users willing to use ContigScape for non-academic purposes should contact the corresponding author for details.

## Abbreviations

NGS: Next-generation sequencing; HTS: High throughput sequencing; CRS: Contig relationship scape.

## Competing interests

The authors declare that they have no competing interests.

## Supplementary Material

Additional file 1 Listing all links of ContigScape, user manual and test datasets. Click here for file
