# Supplementary material for: ContigScape: a Cytoscape plugin facilitating microbial genome gap closing
Source: BMC Genomics. 2013 Apr 30;14:289. doi: 10.1186/1471-2164-14-289 (PMC3651407; doi:10.1186/1471-2164-14-289)
Supplement: Additional file 1 — Listing all links of ContigScape, user manual and test datasets. [file 1471-2164-14-289-S1.docx]

**Additional files**

All additional files can be downloaded at <http://sourceforge.net/projects/contigscape/>.

Cytoscape 2.8.3 can be downloaded at <http://www.cytoscape.org/>

**Additional file 1** –ContigScape, the proposed Cytoscape plugin

**ContigScape.jar**

Download this file at <http://sourceforge.net/projects/contigscape/files/1.0/>. Copy the file into the plugins folder in the Cytoscape-installed folder and run Cytoscape. ContigScape will be automatically loaded to the control panel.

**Additional file 2** ContigScape user manual

usermanual.pdf <http://sourceforge.net/projects/contigscape/files/1.0/>

**Additional file 3** – List of the number of connections among contigs.

[tabbed.txt](http://sourceforge.net/projects/contigscape/files/datasets/tabbed.txt/download) <http://sourceforge.net/projects/contigscape/files/datasets/>

**Additional file 4** – List of all the contigs’ length and coverage.

[tabbedCov.txt](http://sourceforge.net/projects/contigscape/files/datasets/tabbedCov.txt/download) <http://sourceforge.net/projects/contigscape/files/datasets/>

**Additional file 5** – a perl used in converting BLAST output of that using contigs as a query and close-related reference sequence as a database into AGP file.

blast2agp.pl <http://sourceforge.net/projects/contigscape/files/1.0/>

**Additional file 6** – two mate-pair data produced on solexa platform. Please uncompress these packages by typing: tar -zxvf xxxx.tar.gz

[matePairedReads1.fq.tar.gz](http://sourceforge.net/projects/contigscape/files/datasets/matePairedReads1.fq.tar.gz/download) <http://sourceforge.net/projects/contigscape/files/datasets/>

[matePairedReads2.fq.tar.gz](http://sourceforge.net/projects/contigscape/files/datasets/matePairedReads2.fq.tar.gz/download) <http://sourceforge.net/projects/contigscape/files/datasets/>

**Additional file 7** – a fasta file, containing dozens of contigs regards as a reference for ‘*.fq’ file

[matePairedContigs.fas](http://sourceforge.net/projects/contigscape/files/datasets/matePairedContigs.fas/download)

**Additional file 8** –a perl used in prosessing “*.fq” file and corresponding matePairedContigs.fas with the purpose of gaining a tabbed.txt.

Usage: perl scaffold.pl reads1.fq reads2.fq matePairedContigs.fas result_tab

scaffold.pl <http://sourceforge.net/projects/contigscape/files/1.0/>

**Additional file 9** –a AGP file

[test.agp](http://sourceforge.net/projects/contigscape/files/datasets/test.agp/download) <http://sourceforge.net/projects/contigscape/files/datasets/>

**Additional file 10** – Three assembled genome data though ‘Newbler Assembler’ developed by Roche 454. Please uncompress these packages by typing: 7z x 454Contigs.ace.7z , 7z x PO82454Contigs.ace.7z or uncompress by other 7-Zip tools.

tar –zxcf 454Contigs.ace.tar.gz

[454Contigs.ace.7z](http://sourceforge.net/projects/contigscape/files/datasets/454Contigs.ace.7z/download) <http://sourceforge.net/projects/contigscape/files/datasets/>

[PO82454Contigs.ace.7z](http://sourceforge.net/projects/contigscape/files/datasets/PO82454Contigs.ace.7z/download) <http://sourceforge.net/projects/contigscape/files/datasets/>

[454Contigs.ace.tar.gz](http://sourceforge.net/projects/contigscape/files/datasets/454Contigs.ace.tar.gz/download) <http://sourceforge.net/projects/contigscape/files/datasets/>
